# 1484. Hepatitis C Virus Infection and Cure by Priority Population Among New York City Ryan White HIV/AIDS Program Part A Clients

**DOI:** 10.1093/ofid/ofad500.1320

**Published:** 2023-11-27

**Authors:** Jelani Cheek, Tigran Avoundjian, Tristan D McPherson, Mary Irvine

**Affiliations:** New York City Department of Health and Mental Hygiene, New York, New York; New York City Department of Health and Mental Hygiene, New York, New York; New York City Department of Health and Mental Hygiene, New York, New York; NYC Department of Health and Mental Hygiene, New York, New York

## Abstract

**Background:**

Hepatitis C virus (HCV) infection is a common comorbidity among people with HIV (PWH). HCV-related liver disease is the second leading cause of death among PWH. To identify opportunities to improve service integration and health equity, we examined HCV infection and cure among priority populations identified for New York City (NYC) Ryan White HIV/AIDS Program Part A (RWPA) service delivery.

**Methods:**

Using a merge of NYC HIV and HCV surveillance data with RWPA program data, we identified RWPA clients receiving services from 01/2019–12/2022. We categorized clients into priority populations: Black or Latina cisgender women; Black or Latino men who have sex with men (MSM); transgender, intersex, gender non-conforming, or non-binary (TIGNCNB) people; people who inject drugs (PWID); and people aging (age >50) with HIV (PAWH). We measured active HCV infections (any positive RNA test during analysis period), and HCV cure­ (negative RNA result on the most recent HCV test >12 weeks after most recent positive RNA result obtained during the analysis period). Using logistic regression, we compared HCV prevalence and HCV cure by population, adjusting for potential confounders.

**Results:**

Among 15,702 RWPA clients, 15% (n=2,278) had active HCV infection, with 70% (n=1,596) achieving cure. 53% of clients were PAWH, 36% were Black/Latino MSM, 26% were Black/Latina cisgender women, 5% were TIGNCNB people, and 2% were PWID. PWID had the highest proportion of HCV infection (38%), and PAWH had the highest cure proportion (73%) (Table 1). After adjusting for potential confounders, PWID and PAWH had higher odds of HCV infection compared to other clients [adjusted odds ratio (aOR): 4.37, 95% CI: 3.41, 5.59 for PWID; aOR: 4.05, 95% CI: 3.61, 4.54 for PAWH]. Black/Latino MSM and Black/Latina cisgender women had lower odds of infection (aOR: 0.79, 95% CI: 0.71, 0.88 and aOR: 0.60, 95% CI: 0.53, 0.68, respectively) compared to other clients. Among clients with HCV, PWID had lower odds of cure compared to non-PWID (aOR: 0.58, 95% CI: 0.39, 0.85; Table 2).

Table 1, Part 1

**Figure:**
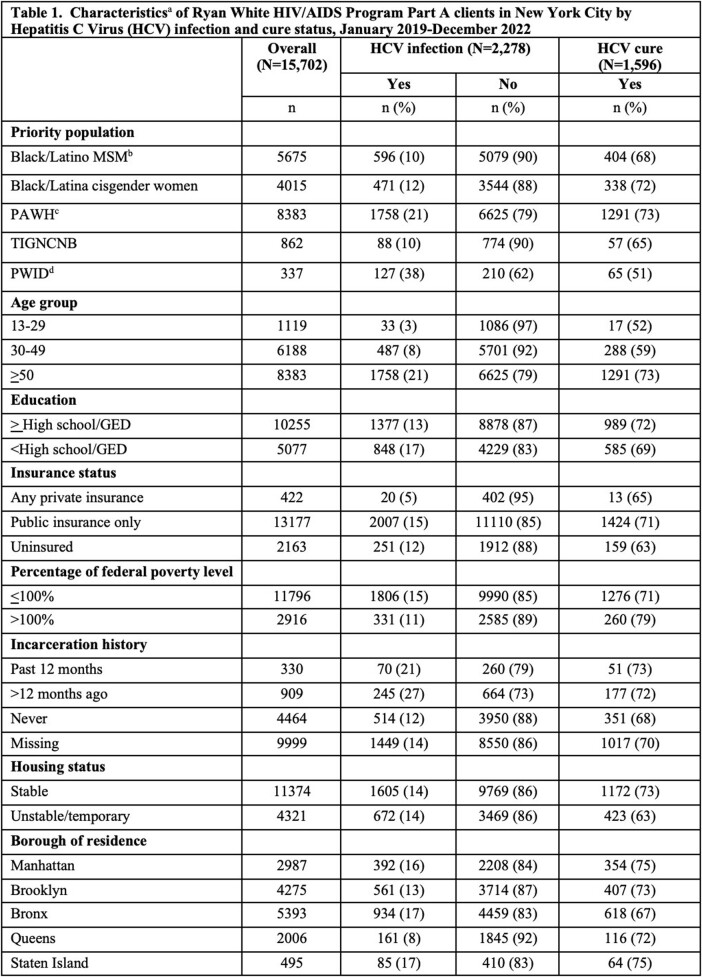

Table 1, Part 2

**Figure:**
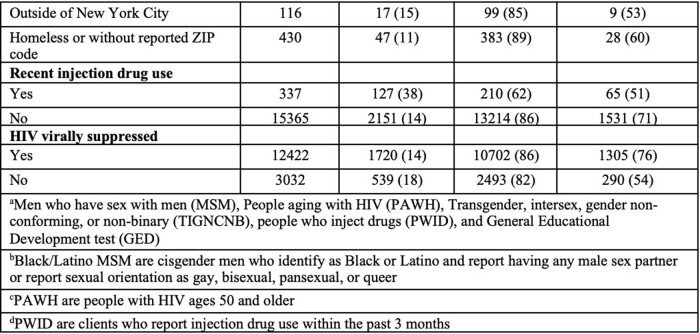

**Figure:**
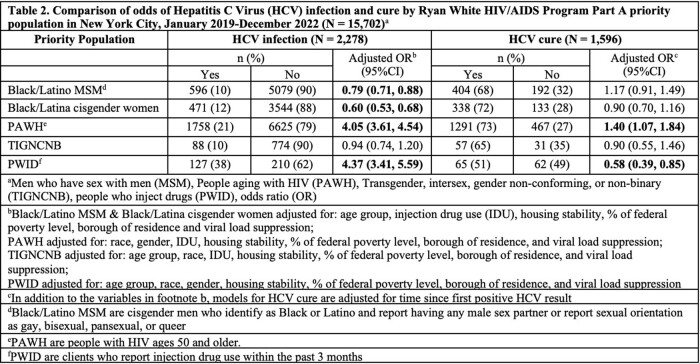

**Conclusion:**

These findings – particularly the low odds of HCV cure among PWID, who had the highest odds of infection – highlight gaps that can inform resource allocation and targeted integration of HIV/HCV care.

**Disclosures:**

**All Authors**: No reported disclosures

